# *gro*EL gene-based molecular detection and antibiogram profile of *Riemerella anatipestifer* from duck in Bangladesh

**DOI:** 10.5455/javar.2022.i637

**Published:** 2022-12-31

**Authors:** Alamgir Hasan, Palash Bose, Mst Tachhlima Aktar, Zobayda Farzana Haque, Mohammad Rafiqul Islam, Muhammad Tofazzal Hossain, Mahbubul Pratik Siddique

**Affiliations:** 1 Department of Microbiology and Hygiene, Bangladesh Agricultural University, Mymensingh, Bangladesh; 2 Department of Nutritional Sciences, College of Human Sciences, Texas Tech University, Lubbock, TX, USA; 3 Livestock Division, Bangladesh Agricultural Research Council, Dhaka, Bangladesh

**Keywords:** groEL gene, duck, antibiogram, Riemerella anatipestifer

## Abstract

**Objectives:**

This study was designed to detect *Riemerella anatipestifer* through polymerase chain reaction (PCR) from duck farming areas of the Mymensingh and Sylhet divisions and to determine the antibiogram profile of the PCR-positive isolates using the disc diffusion method.

**Materials and Methods:**

Fifty two samples were collected, comprising clinically sick (32 ducks) and dead ducks (20). PCR confirmation was accomplished, and consistent findings were observed, employing *R. anatipestifer* *gro*EL (271-bp) gene as appropriate molecular markers. For further clarification, see *R. anatipestifer *specific PCR assay (546-bp) and *gyr*B-based PCR (162-bp) were also done. The disc diffusion method was followed for the antibiotic susceptibility test of the isolates against commonly used antibiotics.

**Results:**

A total of 21 samples, 8 from clinically sick birds and 13 from dead birds, showed positive results in both conventional and molecular assays out of 52 samples. High occurrences were found in oropharyngeal swabs from sick ducks and the liver and heart from dead ducks. Antibiotic susceptibility testing revealed that the isolates were 100% resistant to penicillin G, cefradine, streptomycin, neomycin, gentamycin, meropenem, and erythromycin, but 100% sensitive to ­cotrimoxazole, florfenicol, and levofloxacin.

**Conclusion:**

For diverse duck-populated areas in Bangladesh, this study shows the severity of *R. anatipestifer* infection among ducks.

## Introduction

*Riemerella anatipestifer* infection, previously known as New Duck disease, infectious serositis, or duck septicemia, is an enzootic bacterial disease that causes significant capital losses, particularly in duck farms, due to moderate to high mortality, lower growth performances, increased disapprobation, and expensive treatment expenditures [1]. The causative agent, *R. anatipestifer*, belongs to the family Flavobacteriaceae, though it has recently settled under the family Weeksellaceae [2]. The bacterium is Gram-negative, bipolar, and short-rod revealed through different staining techniques, and also negative for motility, spore formation, and hemolytic activities [3,4]. Throughout the world, the disease is regarded as a financially significant disease [4,5].

The ducklings between 1 and 8 weeks of age are highly susceptible to this disease, and mortality is greater at 4 and 8 weeks, which agrees with Sarker et al. [6]. Moreover, the morbidity and mortality rate in ducklings and adult ducks vary to a greater extent between different age groups [7] and regions [8], and the mortality rate can range from 5% to 75% [9,10] or up to 95% [11]. Serotyping investigations revealed at least 21 serotypes of *R. anatipestifer* in different countries [12–14],and serotypes 1 and 2 are the most pathogenic. Though no significant cross-protection has been reported against *R. anatipestifer* infection [15], regular vaccination, along with duck plague and duck pasteurellosis, may reduce the incidence. Interestingly, *R. anatipestifer* has greater phenotypic similarities with *Pasteurella multocida,* and due to the similarities, both morphologically and culturally, it is sometimes difficult to isolate the organism through conventional methods in the laboratory [16,17].

Ducks and duck farming are the integral part of agricultural economies worldwide, constitute the major part of the industry among poultry species, and are considered next to chicken [18]. The global duck population will be nearly 1.15 billion (*Anas *spp*.*) in 2020, with the Asia continent alone contributing 89% (1.0 billion) of the total duck population [19]. Among Asian countries, China, Indonesia, Vietnam, India, and Bangladesh are well recognized for having the largest duck population [19]. In Bangladesh, the latest update, 597.16 lakh ducks in 2019–2020, proves the continuous rise of duck production compared to the 577.52 in 2018–2019 [20]. At the same time, veterinarians’ history shows that every year, a large amount of economic loss occurs to the marginal farmer due to incorrect diagnoses of various diseases in ducks. *Riemerella anatipestifer *is one of the most severe infections among those diseases.

The disease, Riemerellosis, was first recorded by Mustafa et al. [21] and in a further study by Haque [22], based on cultural and biochemical characteristics, as *Pasteurella anatipestifer*. Subsequently, Sarker et al. [6] performed the molecular characterization of *R. anatipestifer* based on a novel polymerase chain reaction (PCR) assay designed by Kardos et al. [17] for a more confirmatory diagnostic assay. They recorded 35%–65% mortality in the year 2013–2014. Unfortunately, no antibiogram study has been performed yet in Bangladesh against *R. anatipestifer *in ducks. Records say that there are very few effective antibiotics working against this disease in Bangladesh. Despite massive economic losses in Bangladesh, particularly in the Netrokona district of the Mymensingh division and the Sunamgang district, there is no record of a definitive diagnostic assessment from 2014 to 2021.

In this study, the pathogenic organisms were identified based on *gro*EL gene-based PCR, followed by species-­specific gene- and gyrase B-encoding (*gyr*B) gene-based PCR for confirmatory detection approval from the previously published reports [3,17]. Moreover, the antibiogram of *R. anatipestifer *was determined through Kirby Bauer disc diffusion, a rapid and cost-effective method to reduce mortality in the flock. The *gro*EL gene, which has several highly conserved regions, has been explored as a reliable molecular marker for bacterial genus and species identification [23,24]. Yushan et al. [25] and Siddique et al. [26] reported *gro*EL gene superiority regarding heterogeneity, even more than other house-keeping genes such as *16S* and *23S rRNA* genes. The *gro*EL gene encodes a heat shock protein (HSP) known as heat shock protein 60 (HSP60) or 60 kDa chaperonin [23,25]. Han et al. [27]stated that g*ro*EL sequence is highly conserved (over 97.5% identity) and present in all *R. anatipestifer* serotypes.

This study was designed to isolate and identify *R. anatipestifer* from clinically affected and dead ducks from different locations in the Netrokona district and Sunamgang district, under Mymensingh division and Sylhet division, respectively, through conventional (cultural, morphological, and biochemical tests) and molecular detection methods (*gro*EL gene-based PCR), as well as the antibiogram profile determination using the disc diffusion method.

## Materials and Methods

### Sampling and sampling areas

**Figure 1.**
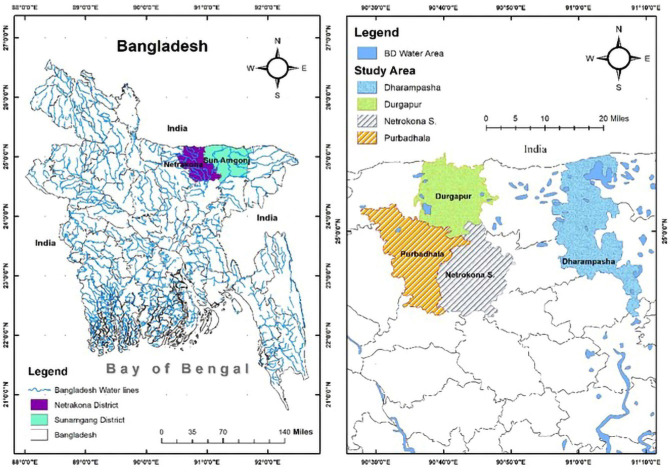
This study area map. This is created with ArcGIS version 10.3 (ESRI, Redlands, CA).

The suspected duck samples were collected from field outbreaks in different areas of Mymensingh division (Netrokona district; Netrokona Sadar; Durgapur; and Purbadhala Upazila) and Sylhet division (Sunamgang district; Dharampasha Upazila) in Bangladesh (Fig. 1). The clinically affected and dead ducks, aged 1.5–2 months, were sampled aseptically in the Upazila Veterinary Hospital of respected Upazila and the Department of Microbiology and Hygiene at Bangladesh Agricultural University (BAU), Mymensingh, after carrying the dead birds through an ice box. All the suspected birds showed clinical signs such as tremors of the head and neck, paddling their legs, incoordination, circular movement, mild coughing and sneezing, and diarrhea. Fifty-two birds (ducks) were sampled for bacteriological studies. In the laboratory, ocular swabs and oropharyngeal swabs (from affected live birds), as well as liver, heart, and lung (from dead birds), were collected from each bird aseptically into the Luria Bertoni (LB) broth. The desired samples were collected at Upazila Veterinary Hospital in a zipper bag and transported through an ice box.

### Bacterial isolates

After the primary enrichment in LB (Hi-Media, Mumbai, India) broth, the samples were then cultured in Nutrient Agar (Hi-Media, Maharashtra, India), Tryptic Soy (TSA) Agar (Hi-Media, Mumbai, India), MacConkey (Hi-Media, Mumbai, India) agar, Eosin Methylene Blue (EMB) agar (Hi-Media, Mumbai, India), and Salmonella-Shigella (SS) agar (Hi-Media, Mumbai, India). Furthermore, suspected colonies were seeded on 10% Bovine Blood (BB) agar and 10% Duck Blood (DB) agar on the TSA base. The bacterial cultures were incubated both micro-aerobically in a candle jar and a normal incubator at 37°C for 24 h. However, the culture plates were incubated in a 5% CO_2_ incubator at 37°C for 24 h. The single pure colony found in the subculture was used for Gram stain to identify the morphology of *R. anatipestifer*. Indole production, the Methyl-Red (MR) test, the Voges-Proskauer (VP) test, H_2_S production, the Oxidase, and Catalase tests, and the sugar fermentation test have been used to confirm *R. anatipestifer*. The Motility Indole Urease (MIU) (Hi-Media, Mumbai, India) test was also performed to observe the motility of the organism. As a positive control, *Pseudomonas aeruginosa* was used to compare whether *R. anatipestifer* is motile or non-motile. 

### Genomic DNA extraction

The DNA of the isolates was extracted by the boiling and throwing method, according to Siddique et al. [26], with little modification. In brief, 1 ml of cultured broth was placed in an Eppendorf tube and centrifuged at 1,000 rpm for 3 min before discarding the supernatant and mixing it with 200 ml of distilled water. The mixture was vortexed and kept in the ice for ice stock after being boiled in hot water for 10 min. Final centrifugation for 3 min was done at 10,000, followed by supernatant collection. The quantity and quality of all the DNA were measured using Nanodrop™ (Thermo Fisher Scientific, USA).

### PCR amplification R. anatipestifer groEL gene

**Figure 2.**
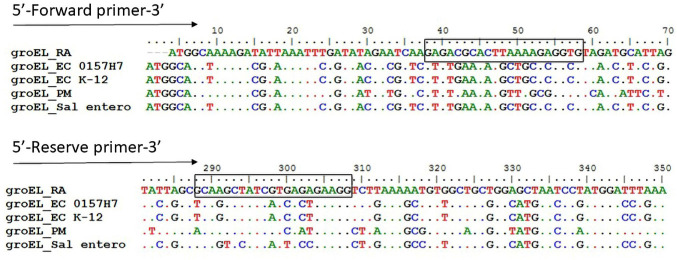
Comparison of *gro*EL primer sequences of *R. anatipestifer* with *E. coli* strain 0157H7, *E. coli* K-12, PM = *P. multocida* and *Salmonella enterocolitis*. The alignment is created by BioEdit sequence alignment software. Box ­indicate the forward and reverse nucleotides sequences for *R. anatipestifer* *gro*EL gene.

The primers for detecting the *gro*ELgene in the genome of *R. anatipestifer* were designed by comparing all possible *gro*ELsequences related to *R. anatipestifer* such as *Escherichia coli*, *P. multocida*, and *Salmonella *spp*.* The forward (RA-groEL-F 5ʹ-GGG AGA CGC ACT TAA AAG AGG TG-3ʹ) and reverse (RA-groEL-R 5ʹ-CCT TCT CTC ACG ATA GCT TGC-3ʹ) primers were designed for PCR amplification (Table 1). The nucleotide sequence of *R. anatipestifer* was achieved from the GenBank of the National Center for Biotechnology Information by using the Nucleotide basic local alignment search tools (BLASTn) search. The Fast allignment sequence test for application (FASTA) sequence of the nucleotide was analyzed by BioEdit Sequence Alignment software for oligonucleotide sequences (Fig. 2). Potential oligonucleotide primers were modeled and synthesized commercially by Biotech, Seoul, Korea. The thermal profile of the *gro*EL gene-based PCR reactions was comprised of 5 min of initial denaturation at 95°C, then 35 cycles of denaturation (94°C for 30 min), annealing (60°C for 30 sec), and extension (72°C for 30 sec), followed by 10 min of final extension at 72°C. The PCR end products were analyzed and visualized as described in the above protocol.

To determine the specificity of the *gro*EL gene-based PCR amplification of *R. anatipestifer*, phenotypically or clinically similar disease-causing bacterial agents were compared, *viz.*, isolates of *P. multocida*, *E. coli*, and *Salmonella typhimurium*. The bacterial isolates were collected from the storage of relevant laboratory repositories (stored at −80°C) at the Department of Microbiology and Hygiene, BAU, Mymensingh. After thawing at room temperature, all the isolates were cultured in nutrient broth for 24 h at 37°C, followed by DNA extraction using the boiling method as previously described.

### PCR amplification R. anatipestifer species-specific and gyrB gene

The bacterial DNA (on average, concentration 130 ng/μl, purity 2.10) was standardized for the *Riemerella *species-specific primers and the *gyrB* gene (Table 1). The total volume of the PCR reaction mixture was 25 μl including 5 μl DNA template, 1 μl forward and reverse primer, 12.5 μl PCR master mix, 2X (Promega, Madison, WI), and 5.5 μl nuclease-free water. The PCR reactions were conducted according to the previously published protocol by Rubbenstrothet al.[3]. In brief, a single cycle of initial denaturation at 94°C for 2 min is followed by 35 cycles of denaturation at 94°C for 30 sec, annealing at 54°C for 30 sec, extension at 72°C for 30 sec, and a single cycle of final extension at 72°C for 10 min. The PCR end products were analyzed using a 1.5% agarose gel. After completion of electrophoresis, the gel was stained via soaking in ethidium bromide solution (approximate concentration 0.2–0.5 μg/ml) for 10 min and finally visualized using a UV transilluminator.

### Antibiotic susceptibility of R. anatipestifer

In this study, the antibiogram test was performed to determine the antibiotic susceptibility of commonly used antibiotics by the disc diffusion method at the field level. For the antibiotic susceptibility test, all the isolates were cultured in LB broth for 4 h at 37°C in a shaking incubator, and the turbidity was adjusted to the 0.5% McFarland standard. The antibiotic discs were placed on the inoculated surface of Muller-Hinton agar (Hi-Media, Mumbai, India) and incubated for 18 h at 37°C temperature to observe the zone diameter. The antibiotic discs, including penicillin G (P 10), meropenem (MEM 10), colistin (CL 10), amikacin (AK 30), neomycin (N 30), cotrimoxazole (COT 25), erythromycin (E 15), azithromycin (AZM 15), streptomycin (S 10), kanamycin (K 30), Gentamycin (GEN 10), cefuroxime (CXM 30), ceftriaxone (CTR 30), cefradine (CH 25), nalidixic acid (NA 30), chloramphenicol (C 30), florfenicol (FFC 25), ciprofloxacin (CIP 5), levofloxacin (LEV 5), and novobiocin (NV 30) were used in this study. After 18 h of incubation at 37°C, the diameter of the “zone of inhibition” was calculated and clarified based on the guidelines from Clinical and laboratory standards institute (CLSI) documents M100-S21 and VET01S (CLSI, 2013, 2015) mentioned in Gyuris et al. [28]. The rest were interpreted based on *P. multocida* zone diameter due to incomplete reference data in the CLSI guidelines for *R. anatipestifer*. 

### Statistical analysis

All data, including this manuscript, are analyzed using Microsoft Word and Excel software version 16.

**Figure 3.**
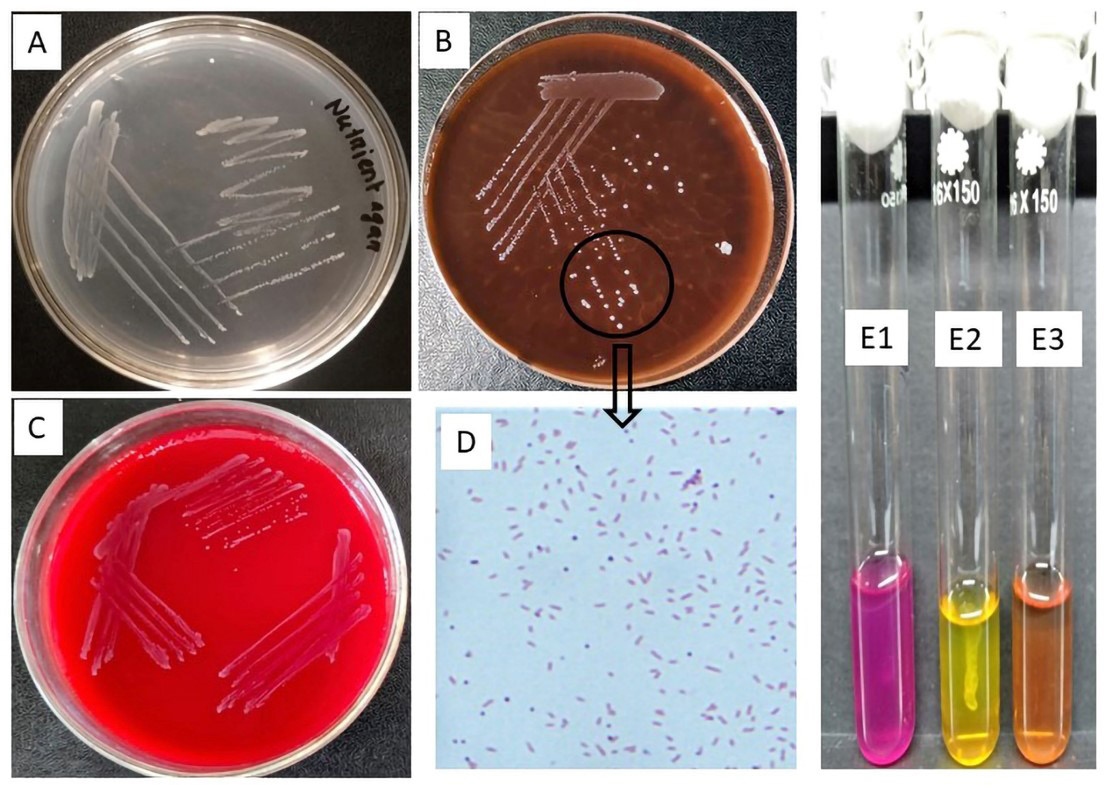
Nutrient agar (Small rounded pale white colony), B. 10% Duck Blood agar (Small, shiny, rounded and pearl like colony), C. 10% Bovine Blood agar (Small, rounded non β-hemolytic and shiny white color), D. Gram stain (short-rod shaped coccobacilli), and E. Molity test of *R. anatipestifer* (E1. Motile *Pseudomoas aeruginosa* as positive control; diffuse bacterial growth, E2. Non-motile *R. anatipestifer*; single line growth of bacteria, and E3. Negative control).

## Results

All the affected ducks showed clinical signs: tremors of the head and neck, paddling their legs, incoordination, circular movement, mild coughing and sneezing, and diarrhea. Hemorrhage and congestion on the liver were observed, as perihepatitis with a normal size liver, pericarditis with a white lychee-like covering on the heart, and hemorrhage on the trachea and lung. A total of 21 samples, 8 from live birds and 13 from dead, showed positive results conventionally and by molecular assay out of 52 samples (Tables 2 and 3).

### Bacterial culture and staining

The bacterial isolates grew smoothly in LB broth with diffuse turbidity and nutrient broth at 37°C for 24 h in both microaerophilic and aerophilic conditions. In the case of BB agar, the bacterial colonies were small, greyish-white, rounded, and moist, and no hemolytic activity was observed (Fig. 3B). Besides, on DB agar, the colonies were white, glistening, small, and non-hemolytic, with 24 h incubation at 37°C both the microaerophilic and aerophilic incubators (Fig. 3B). Similarly, the same colonies were found on blood agar with 5% CO_2_ at 37°C for 24 h. Moreover, the bacteria appeared smooth, circular, and grayish on TSA (Fig. 3A). In contrast, there was no growth on MacConkey, S-S, or EMB agar. Gram stain revealed Gram-negative Coccobacilli that were small rod-shaped (Fig. 3D).

### Motility and biochemical test

The MIU test revealed that the organisms were non-motile (Fig. 3E) and urease and indole negative. The other biochemical tests, such as oxidase and catalase, and MR were positive; H_2_S and VP tests were negative. *Riemerella anatipestifer *isolates fermented Dextrose, Maltose, and Sucrose with the formation of acid and gas; however, they could not ferment lactose and Mannose.

### Specificity of groEL gene

**Figure 4.**
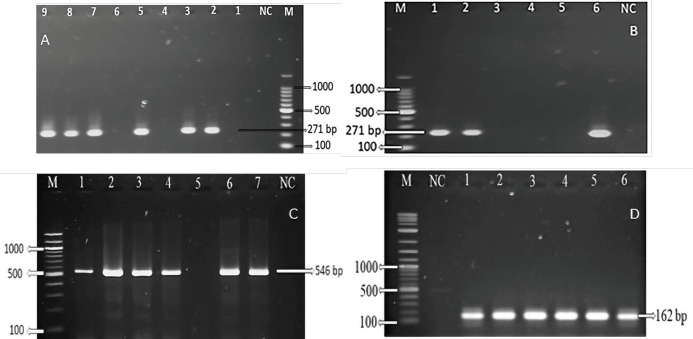
*A. Riemerella anatipestifer gro*EL gene at 271-bp amplicon size, M = 100-bp Marker; Lane 2, 3, 5, 7, 8, 9 = Positive isolates; Lane 1, 4, 6 = Negative isolates, NC = Negative control, B. *groEL *gene at 271-bp amplicon size, M = 100-bp Marker, NC = Negative control, Lane 1, 2, 6 = *gro*El positive isolates, Lane 3 = *E.coli*, Lane 4 = *P. multocida*, Lane 5 = *Salmonella* spp., C. *R. anatipestifer* species-specific gene at 546-bp amplicon size, M = 100-bp Marker; Lane 1, 2, 3, 4, 6, 7 = Positive isolates, 5 = Negative isolates, NC = Negative control, D. *gyr*B gene at 162-bp amplicon size, M = 1kb Marker, NC = Negative control, Lane 1-6 = positive isolates.

All the species-specific positive bacterial samples revealed a 271-bp amplicon size according to the *gro*EL gene (Fig. 4A) of *R. anatipestifer* at 60°C annealing temperatures. However, it was tested for several annealing temperature ranges from 58°C to 61°C. However, the mentioned temperature of 60°C has been fixed for the target amplicon size. For specificity of the *gro*EL gene, the designed primer was tested using the DNA of *E. coli*, *P. multocida*, and *Salmonella* spp. (from laboratory storage). The primer revealed no band size in the case of *E. coli*, *P. multocida*, and *Salmonella* spp. (Fig. 4B). 

### Riemerella anatipestifer species-specific gene and gyrB gene

All the positive* R. anatipestifer* isolates showed a positive band at a 546-bp amplicon size by *R. anatipestifer* species-specific gene (Fig. 4C) with the variations of annealing temperature 54°C to 60°C, and 54°C produced an optimum result. Furthermore, the *gyr*B gene (Fig. 4D) revealed a 162-bp amplicon size, according to Udayan et al. [16]. 

### Antibacterial susceptibility of R. anatipestifer

**Figure 5.**
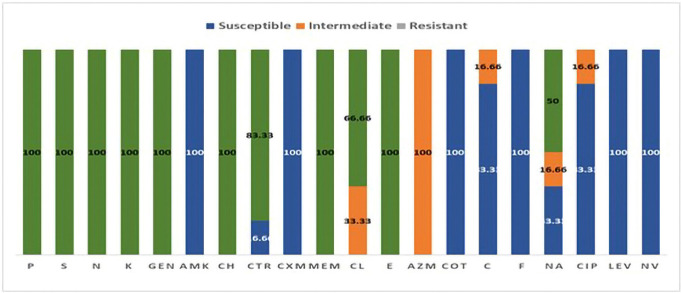
The antibiotic susceptibility ranges of *R. anatipestifer*, P = penicillin, S = streptomycin, N = neomycin, K = kanamycin, GEN = gentamycin, AMK = amikacin, CH = cefradine, CTR = ceftriaxone, CXM = cefuroxime, MEM = meropenem, CL = colistin, E = erythromycin, F = florfenical, AZM = azithromycin, COT = cotrimoxazole, NA = nalidixic acid, CIP = ciprofloxacin, LEV = levofloxacin, NV = novobiocin.

All the positive isolates were tested for antibiotic susceptibility with different groups of antibiotics. Among them, large groups of antibiotics such as beta-lactams (penicillin G, cefradine), aminoglycosides (streptomycin, neomycin, and gentamycin), penems (meropenem), and macrolides (erythromycin) elicited 100% resistance. In comparison, cefuroxime presented 80.95% resistance among *R. anatipestifer* isolates, and ceftriaxone was found susceptible to *R. anatipestifer*. Subsequently, sulfonamides (cotrimoxazole), phenicols (florfenicol), and quinolones (levofloxacin) showed 100% susceptibility to all isolates. The highest percentage of intermediate resistance pattern was found in colistin, 33.33%, and only 4.76% in ciprofloxacin (Fig. 5). 

## Discussion

*Riemerella anatipestifer* infection in ducks has become an emerging problem in many countries. In Bangladesh, a huge number of ducks are died every year due to diagnostic errors with other organisms like *Pasteurella *spp*.*, *Salmonella *spp*.,* *E. coli*, duck plague, duck viral hepatitis, and avian influenza due to their phenotypic similarities, which was also observed in other studies [1,17,29].

In our study, we conducted a short survey on susceptible age groups, morbidity, mortality, clinical features, vaccination, and treatment commonly used by veterinary surgeons (VS) in the respected Upazila. The ducklings at 2–8 weeks of age were highly susceptible to this disease, and maximum mortality was found at 4–8 weeks of age in mid-May to July, according to the statement of farmers and VS. The maximum morbidity and mortality were observed during June to July at the 8–10 weeks of age group by Sarker et al. [6], whereas the outbreak was also reported during the summer [22]. The mortality rate in ducklings was higher than that in adult ducks, which agrees with Doley et al. [30]. Based on the farmer’s history, the survey found that morbidity and mortality were 75%–80% and 40%–45%, respectively. In a previous study, researchers showed the mortality rate to be 35%–65% in Bangladesh [6]. The morbidity and mortality vary depending on the age (below 8 weeks), co-infection (*E. coli*, *Salmonella *spp., *P. multocida,* and so on), and other stress factors (environment, climate conditions, and nutrition), which are up to 75% [31–33]. However, all the affected ducks were found showing the clinical signs—tremors of the head and neck, paddling their legs, incoordination, circular movement, mild coughing, sneezing, and diarrhea—and the owners of the affected flocks claimed the same observations, which support the recent study [5]. Postmortem changes included hemorrhage and congestion on the liver, perihepatitis with normal liver size, pericarditis with a white lichee-like covering on the heart, and hemorrhage on the trachea and lung. Sarker et al. [6] reported widespread hemorrhage and congestion in the body cavity, gray-colored necrotic foci on the liver, an enlarged kidney, and one patient hemorrhage. One study by Chikuba et al. [34] found whitish, gelatinous, and fibrinous exudates are covering the heart and liver surfaces.

**Table 1.** The oligonucleotides for identification of *R. anatipestifer.*

Target gene	Sequences	Amplicon size	Reference
*groEL*	F- GGGAGACGCACTTAAAAGAGGTG	271 bp	This study
	R- CCTTCTCTCACGATAGCTTGC		
*gyrB *	F-GGCTAAGGCAAGACAAGCTG	162 bp	[16]
	R-GCAGTTCCTCCTGCAGAGTC		
Species-specific	F-TTACCGACTGATTGCCTTCTAG	546 bp	[17]
	R-AGAGGAAGACCGAGGACATC		

bp-base pair.

In this study, we collected ocular and oropharyngeal swabs from affected live birds and liver, heart, and lung from dead ducks for a chronological study. We used LB broth, nutrient agar, TS agar, EMB agar, SS agar, MacConkey agar, BB agar, and DB agar to isolate *R. anatipestifer.* The occurrence of *R. anatipestifer* in ocular swabs was low at only 10%, whereas oropharyngeal swabs were 30% in affected live birds. In a previous study, it was recorded that *R. anatipestifer* is the common flora for pharyngeal and laryngeal swabs [35,36]. In addition, in the case of dead ducks, the occurrence was 35% and 25% for the heart and liver, respectively.

With a 24 h incubation at 37°C in both microaerophilic and aerophilic incubators, the bacterium in BB agar produced a small, gray, non-hemolytic appearance and a pearl-like appearance on DB agar. Similarly, the same colonies were found on blood agar with 5% CO_2_ at 37°C for 24 h. BB agar at a 10% level has been reported to be useful for the primary isolation of the organism at 37, in which an atmosphere enriched with 5%–10% CO_2_ for 24 h was used for the growth [31,37]. The organisms were also grown on 10% sheep blood agar plates in an atmosphere enriched with 5% CO_2_, as described by Priya et al. [37]. Recently, Majhi et al. [8] concluded that the cultural characteristics on various mediums were like small, non-hemolytic colonies on blood agar; smooth, grey, glistening, and dewdrop-like colonies on nutrient agar; and the bacteria grew but did not produce metallic sheen on EMB agar. In contrast, we found no growth on EMB agar, MacConkey agar, which agreed with Pala et al. [1], Shancy et al. [4], Surya et al.[7], and SS agar. Moreover, the bacteria appeared smooth, circular, and grayish on TSA.

**Table 2.** The prevalence of *R. anatipestifer* in different areas.

Source	Total sample	Nature of the sample	No. of the sample	No. of positive isolates	Prevalence (%)	
					Live and dead	Total
Durgapur	22	Sick	15	5	33.3	54.54
		Dead	7	7	100	
Purbadhala	14	Sick	7	1	14.28	42.85
		Dead	7	5	71.42	
Netrokona sadar	10	Sick	6	0	0	0
		Dead	4	0	0	
Sunamgang	6	Sick	5	2	40	50
		Dead	1	1	100	

**Table 3.** The occurrence of *R. anatipestifer* from different samples.

Nature of samples	Total sample	No. of positive result	Occurrence (%)
Clinically affected ducks (32)			
Ocular swab	32	2	10
Oropharyngeal swab	32	6	30
Subtotal	64	8	40
Dead ducks (20)			
Liver	20	7	35
Heart	20	5	25
Lung	20	0	0
Subtotal	60	13	55
Grand total = 52		21	40.38

Gram stain revealed Gram-negative coccobacilli, a short rod-shaped organism, and Pillai et al. and Heba et al. [38,39] found similar results but were larger in size than *P. multocida* [7]. For a more accurate diagnosis, conventional biochemical tests such as Indole production, MR test, VP test, H_2_S production, Oxidase, and Catalase tests, sugar fermentation (glucose, lactose, maltose, mannitol, dextrose, and sucrose) tests were used and produced similar results as mentioned in Sarker et al. [6]: fermentation of dextrose Similarly, oxidase test positivity and H_2_S negativity were mentioned by Surya et al. and Shancy et al. [4,7].

The PCR is considered the gold standard in terms of specificity, sensitivity, and reliability for detecting microbial causal agents of diseases [40]. In this study, PCR was performed targeting the *gro*EL gene for specific confirmation of *R. anatipestifer* isolates and finds a specific and consistent amplicon at 271-bp. In addition, we applied a *Riemerella* species-specific gene designed by Kardos et al. [17] and the *gyr*B gene primarily. A band of 546-bp size was observed by species-specific primers at annealing temperature 54, according to Rubbenstroth et al. [3]. Kardos et al. [5], Kardos et al. [17], Soman et al. [40] and Hazarika et al. [5]stated the same amplicon size in their studies. Interestingly, the *gyr*B gene was a more accurate, sensitive, and specific biological marker for *R*. *anatipestifer* detection at the molecular level [16]. Furthermore, Yamamoto and Harayama [41] demonstrated that *gyr*B is found in all bacterial strains. In our study, all bacterial isolates showed 162-bp amplicons using the same conditions found in Udayan et al. [16]. However, *Gro*EL is a member of the molecular chaperon family *Gro*E system, together with *gro*ES [42]. The *gro*EL gene-based PCR assays have already been established by various researchers in the last few decades [24,26,43,44]and have proven to be powerful phylogenetic markers [45].

The positive isolates were then tested for antibiotic resistance profiles with different groups of antibiotics. Among them, large groups of antibiotics such as beta-lactams (penicillin G, cefradine), aminoglycosides (streptomycin, neomycin, and gentamycin), penems (meropenem), and macrolides (erythromycin) elicited 100% resistance. In comparison, cefuroxime presented 80.95% resistance among *R. anatipestifer* isolates, and ceftriaxone was found susceptible to *R. anatipestifer*. Subsequently, sulfonamides (cotrimoxazole), phenicols (florfenicol), and quinolones (levofloxacin) showed 100% susceptibility to all isolates. The highest percentage of intermediate resistance pattern was found in colistin, 33.33%, and only 4.76% in ciprofloxacin. Many antimicrobial agents have been used for controlling the infection of *R. anatipestifer *and reducing significant economic losses at the field level through various studies over time*. In vivo* susceptibility testing revealed sensitivity to enrofloxacin, ciprofloxacin, ofloxacin, and neomycin by Hazarika et al. [5], and several studies stated that ciprofloxacin, gentamycin, polymyxin-B, chloramphenicol, norfloxacin, doxycycline, gentamicin, clindamycin, and cefuroxime were sensitive to *R. anatipestifer *[7,8,46]. In contrast, methicillin, sulfadiazine, penicillin-G, metronidazole, erythromycin, oxacillin, polymyxin B, sulfadiazine, cefuroxime, and ampicillin were found resistant to* R. anatipestifer *by Surya et al. and Majhi et al. [7,8]. Moreover, gentamicin, cefazolin [5], penicillin, ampicillin, and tetracycline, were also resistant [46]. Only three antibiotics, streptomycin, lincomycin, and doxycycline, were immediately sensitive to *R. anatipestifer *[5,8]. So, it is clear that *R. anatipestifer* drug resistance profiles changed over time, which is also agreed upon by Zhong et al. [46].

## Conclusion

The overall detection rate of *R. anatipestifer* in ducks was 40.38% (21/52). Multidrug-resistant isolates were prevalent in the study areas, which is alarming for both the sustainable duck industry and public health. Moreover, the *gro*EL gene could be reliably explored with high specificity for detecting *R. anatipestifer* in ducks from field outbreaks. Molecular characterization through sequencing of various house-keeping and other genes, virulence-associated gene detection, pathogenicity study, antimicrobial resistance gene detection, and comparative phylogenetic analysis are considered future research on *R. anatipestifer* in the context of Bangladesh. 

## List of abbreviations

BAU, Bangladesh Agricultural University; BLASTn: Nucleotide basic local alignment search tools; CLSI, clinical and laboratory standards institute; HSP, Heat shock protein; kDa, Kilodalton.
